# Multimodal Brain Connectivity Analysis in Unmedicated Late-Life Depression

**DOI:** 10.1371/journal.pone.0096033

**Published:** 2014-04-24

**Authors:** Reza Tadayonnejad, Shaolin Yang, Anand Kumar, Olusola Ajilore

**Affiliations:** Department of Psychiatry, University of Illinois at Chicago, Chicago, Illinois, United States of America; University of Michigan, United States of America

## Abstract

Late-life depression (LLD) is a common disorder associated with emotional distress, cognitive impairment and somatic complains. Structural abnormalities have been suggested as one of the main neurobiological correlates in LLD. However the relationship between these structural abnormalities and altered functional brain networks in LLD remains poorly understood. 15 healthy elderly comparison subjects from the community and 10 unmedicated and symptomatic subjects with geriatric depression were selected for this study. For each subject, 87 regions of interest (ROI) were generated from whole brain anatomical parcellation of resting state fMRI data. Whole-brain ROI-wise correlations were calculated and compared between groups. Group differences were assessed using an analysis of covariance after controlling for age, sex and education with multiple comparison correction using the false discovery rate. Structural connectivity was assessed by tract-based spatial statistics (TBSS). LLD subjects had significantly decreased connectivity between the right accumbens area (rA) and the right medial orbitofrontal cortex (rmOFC) as well as between the right rostral anterior cingulate cortex (rrACC) and bilateral superior frontal gyrus (bsSFG). Altered connectivity of rrACC with the bsSFG was significantly correlated with depression severity in depressed subjects. TBSS analysis showed a 20% reduction in fractional anisotropy (FA) in the right Forceps Minor (rFM) in depressed subjects. rFM FA values were positively correlated with rA-rmOFC and rrACC-bsFG functional connectivity values in our total study sample. Coordinated structural and functional impairment in circuits involved in emotion regulation and reward pathways play an important role in the pathophysiology of LLD.

## Introduction

As a common psychiatric disorder in older adults, late-life depression (LLD) has a negative impact on the lives of the elderly by adversely affecting cognitive, emotional and somatic aspects of their mental and physical health [1]. LLD is typically defined as the presence of major depressive disorder after age 60–65 and is comprised of early-onset and late-onset subtypes. Early-onset LLD represents the first age of depression onset early in life, in addition to episodes that occur in the geriatric age range, whereas late-onset LLD is defined as new onset depression after age 60 [2]. Different neurobiological etiologies have been suggested in LLD including structural abnormalities due to underlying vascular and neurodegenerative factors, hypothalamo-pituitary-adrenal (HPA) axis dysfunction and neurotransmitter dysregulation [3]. By disturbing the normal function and dynamics of different brain networks, those pathophysiological mechanisms may generate different specific clinical symptoms.

Among the abnormalities associated with LLD, structural alterations, including gray matter volume and shape alterations, and white matter micro- and macrostructural changes in frontolimbic circuitry which have received a great deal of attention and have been widely shown in several structural imaging studies in LLD [1], [4]–[7]. Commonly two imaging approaches are used to investigate the changes in white matter structure and integrity. Macrostructural changes characterized by increased white matter hyperintensities (WMH) volume are identified with magnetic resonance imaging (MRI) [8], [9]. Diffusion tensor imaging (DTI) is used to detect microstructural abnormalities by quantifying the integrity of axon tracts indicated by fractional anisotropy (FA). DTI studies in LLD have typically focused on specific predetermined regions of interest and found FA reductions predominately in the frontal lobe and to a lesser extent in temporal regions [10]–[14].

Neuroimaging studies of LLD have been primarily focused on structural brain alterations and an increasing effort has been made in understanding the mechanism of LLD at the functional network level. Classically, the function of a given network is tested by measuring the level of activity in terms of oxygen or glucose utilization in connected brain regions during the performance of a specific task. Task-based functional magnetic resonance imaging (fMRI) studies in LLD have revealed abnormal activity in several key regions of frontostriatal-limbic circuitry during cognitive and emotion tasks [1]. In frontal regions, altered activity was reported in the dorsolateral prefrontal cortex (dlPFC) during preparation to overcome prepotency and explicit learning tasks [14]–[16], in the ventromedial prefrontal cortex (vmPFC) in response to emotionally negative stimuli [17], and in the bilateral superior frontal gyrus (sFG) and orbitofrontal cortex (OFC) during the stop signal task (SST) [18] in patients with LLD compared to healthy control subjects.

Another method for exploring the dynamics of brain networks is measuring the correlation and synchronicity of activity between brain regions at rest using functional connectivity (FC) analysis [19]. This approach has been used widely in the hope of finding objective reliable biomarkers of pathophysiology, disease and treatment response in different neurological and psychiatric disorders [20]. Methodologically, two main approaches are applied for FC analysis: hypothesis-driven and data-driven. In hypothesis-driven approaches, a “seed” region of interest is selected first and then correlation of activity of that seed with pre-defined region(s) or all brain voxels is measured during the resting state. In contrast, data-driven methods of FC analysis place no emphasis on a specific brain region and the whole brain is investigated for detecting significant correlation patterns. Over the past few years, some FC analysis studies in LLD have been conducted using resting-state fMRI (rs-fMRI) [14], [21]–[25]. These studies have revealed significant correlations between FC measures and the severity of clinical symptoms [14], [26]. Commonly, the hypothesis-driven method was used in those studies and in some studies medicated or remitted individuals were used as participants [22], [23], [25]. Their results have primarily shown abnormal changes in FC between nodes belonging to default mode network (DMN) and cognitive control network (CCN) [21], [24].

The relationship between aforementioned structural changes in LLD and FC alterations remains poorly understood. In healthy individuals, a strong relationship has been demonstrated between anatomical and resting state functional connectivity [27]. In addition, in midlife depression, some studies have reported significant correlations between pathologic changes in gray matter volume or white matter integrity and functional abnormalities in depression-related neurocircuitries [28], [29]. There have only been two preliminary studies that examine the relation between white matter integrity and functional connectivity in restricted pre-defined ROIs in subjects with LLD [24], [30]. None of those studies compared their results with a healthy comparison group.

To our knowledge, no study has explored whole brain rs-fMRI functional connectivity changes in unmedicated and symptomatic patients with LLD. Furthermore, there have been no studies to date that have investigated the relationship between whole brain white matter tract integrity and FC values in patients with LLD compared to healthy control subjects. The purpose of our study was to examine FC alterations using rs-fMRI in association with white matter integrity measured by DTI in unmedicated patients with LLD. We hypothesized that abnormal changes in brain network FC and pathologic alterations in white matter integrity can occur in concert in LLD and relate to symptom severity in unmedicated patients suffering from geriatric depression. We also hypothesized that there will be a significant correlation between structural connectivity assessed by FA and functional connectivity assessed by rs-fMRI in unmedicated elderly with LLD. To test our hypotheses, a data-driven method was applied for FC analysis of rs-fMRI data acquired from unmedicated and symptomatic patients with LLD and a group of healthy comparison subjects. Changes in integrity of whole brain white matter tracts were also tested by applying an automated tract-based spatial statistics (TBSS) method to analyze diffusion tensor imaging (DTI) data [31] obtained from depressed and comparison subjects.

## Methods

We studied 25 subjects 60 years of age and older. Of these, 10 were unmedicated subjects with unipolar major depression (LLD) and 15 were nondepressed comparison subjects (HC). All study subjects were recruited from the local community through advertisements in flyers, newspapers, and radio. The inclusion criteria for all subjects were 60 years of age and older, antidepressant-naive or free of antidepressant use for at least two weeks and no history of unstable cardiac or neurological diseases. Six LLD subjects were treatment-naïve. For the remaining four, there have been varying degrees of antidepressant exposure. One subject started citalopram and bupropion 6–7 years ago and discontinued the medication 6 months prior to study entry. Another subject had been on venlafaxine for 4 years stopped 6 years before study entry. One subject had been on sertraline for unknown duration prior to study entry and the last subject stopped fluoxetine 9 years prior to study entry. The exclusion criteria included: schizophrenia, bipolar or any psychotic disorders; history of anxiety disorder outside of major depressive episodes; history of head trauma or loss of consciousness; history of substance abuse; contraindications to MRI such as metal implants; Mini Mental Status Exam (MMSE) Score ≤ 24. This study was approved by the University of Illinois-Chicago Institutional Review Board, and written informed consent was obtained from each participant in accordance with the Declaration of Helsinki.

All eligible subjects were assessed by a trained research assistant with the Structured Clinical Interview for Diagnostic and Statistical Manual of Mental Disorders, Fourth Edition[32]. The severity of depression was quantified by a board-certified/board-eligible psychiatrist (AK or OA) using the 17-item Hamilton Depression Rating Scale (HAM-D) [33]. At the time of enrollment, depressed subjects met DSM-IV criteria for MDD and required a score of 15 or greater on the HAM-D. Subjects were also administered the Geriatric Depression Scale (GDS) scale as an independent measure of depression severity [34]. The GDS was used for correlation analyses as the HAM-D was the measure used in the determination of subject eligibility for depression.

### MRI Acquisition

Brain MRI data were acquired on a Philips Achieva 3.0T scanner (Philips Medical Systems, Best, The Netherlands) using an 8-channel SENSE (Sensitivity Encoding) head coil. Participants were positioned comfortably on the scanner bed and fitted with soft ear plugs; foam pads were used to minimize head movement. Participants were instructed to remain still throughout the scan. High resolution three-dimensional T_1_-weighted images were acquired with a MPRAGE (Magnetization Prepared Rapid Acquisition Gradient Echo) sequence (field of view: FOV = 240 mm; 134 contiguous axial slices; TR/TE = 8.4/3.9 ms; flip angle = 8^o^; voxel size = 1.1 × 1.1 × 1.1 mm). Resting-state data were acquired with the following parameters: Single-shot gradient-echo EPI sequence, TR/TE  =  2000/30 ms, Flip angle  =  80 degree, EPI factor  =  47, FOV  =  23 × 23 × 15 cm^3^, in-plane resolution  =  3×3 mm^2^, slice thickness/gap  =  5/0 mm, slice number  =  30, SENSE reduction factor  =  1.8, NEX  =  200, total scan time  =  6∶52. Subjects were instructed to keep their eyes close and “not think of anything in particular”. DTI images were acquired using single-shot spin-echo echo-planar imaging (EPI) sequence (FOV = 240 mm; acquired voxel size  =  2.14 × 2.14 × 2.20 mm^3^; reconstructed voxel size = 0.83 × 0.83 × 2.2 mm^3^; TR/TE = 6994/71 ms; flip angle = 90^o^). Sixty seven contiguous axial slices aligned to the anterior commissure–posterior commissure (AC-PC) line were collected in 32 gradient directions with b = 700 s/mm^2^ and one acquisition without diffusion sensitization (B_0_ image). Parallel imaging technique was utilized with factor at 2.5 to reduce scanning time to approximately 4 minutes.

### Data preprocessing

Functional connectivity was measured using the resting-state fMRI toolbox, CONN v.1.2 (http://www.nitrc.org/projects/conn; [35]). Using pre-processing tools from Statistical Parametric Mapping 8 [36], raw EPI images were realigned, co-registered, normalized, and smoothed with a smoothing kernel of 8 mm before analyses. In addition, the artifact detection tool (ART: http://www.nitrc.org/projects/artifact_detect) was used to measure motion artifacts in all subjects. There was no significant difference in composite motion between groups (means ± standard deviation; HC:.275±.142, LLD:.292±.143, p  = .77), nonetheless we controlled for any motion artifacts using realignment parameters detected by ART. The principal components of the white matter and CSF signal were regressed out of the signal using the CompCor method [37]. BOLD signal data was passed through a band-pass filter of.008 to.09 Hz. Using 87 regions of interest (ROIs) defined by the Freesurfer Desikan atlas [38], functional connectivity measures were derived using pairwise BOLD signal averages correlations after Fisher's r-to-z transformations. An 87×87 connectivity matrix or connectome was created and analyzed as part of the second level analyses completed in CONN.

Voxelwise statistical analysis of the FA data was carried out using TBSS (Tract-Based Spatial Statistics)[31], part of FSL [39]. First, FA images were created by fitting a tensor model to the raw diffusion data using FDT, and then brain-extracted using BET. All subjects' FA data were then aligned into a common space using the nonlinear registration tool FNIRT[40], [41], which uses a b-spline representation of the registration warp field [42]. Next, the mean FA image was created and thinned to create a mean FA skeleton which represents the centers of all tracts common to the group. Each subject's aligned FA data was then projected onto this skeleton and the resulting data fed into voxelwise cross-subject statistics.

### Statistical Analysis

Demographic, clinical, and network variables were analyzed for between-group differences using an independent sample t-test for continuous variables and chi-squared test for categorical variables. Levene's test was used to test the equality of variance. FC and FA group differences were analyzed using univariate analysis of covariance with age, sex, and education as covariates. Multiple comparison correction for FC measures at the connection level was conducted using the false discovery rate (FDR), implement in CONN [43]. In CONN, FDR at the connection level takes into multiple comparisons when exploring the entire connectome. Effect sizes for significant FC measures were calculated using the partial eta squared. Given on a priori assumptions based on FC measures, we used an uncorrected significance threshold of p<.001 for TBSS results. Pearson's correlations were used to analyze the relationship between FC measures, FA and depression severity.

## Results

### Subjects

Compared to the comparison group, patients with LLD had a significantly lower mean age (HC: 71.7±6.8 years; LLD: 62.9±2.5 years; t = 4.34, df = 19, p = .001). The mean age of onset for LLD subjects was 39.6±19.9 years of age. 2 of the 10 subjects had late-onset depression (at 60 years of age or older). There were no significant differences between the two groups in gender (HC: 7 males/8 females; LLD: 4 males/6 females; Χ^2^ = .11,df = 1, p  = .74), MMSE (HC: 29.0 ±1.1; LLD: 28.9±1.2; t = .22, df = 23, p = .83) or education (HC: 14.3±2.1 years; LLD: 14.5±2.6 years,;t =  −.25, df = 23, p = .81). As expected, depressed subjects scored significantly higher on both measures of depression severity (HAM-D, HC: 1.6±1.6., LLD: 19.6±3.5; t = −17.5, df = 11.71, p<.001; GDS, HC: 2.6±3.1, LLD: 22.2±4.6, t = −11.86, df = 20, p<.001) (Table 1).

**Table 1:** Demographic and clinical characteristics (MMSE: Mini Mental Status Examination; HAM-D: Hamilton Rating Scale for Depression; GDS: Geriatric Depression Scale).

	HC (n = 15)	LLD (n = 10)	P values
Age	71.2±6.8	62.9±2.5	0.001^a^
Sex (M/F)	7/8	4/6	0.11^b^
Education	4.3±2.1	14.5±2.6	0.81^a^
MMSE	29.0± 1.1	28.9 ±1.2	0.83
HAM-D	1.6±1.6	19.6±3.5	<0.001 a
GDS	2.6±3.1	22.2±4.6	<0.001^a^

a The P values were obtained by sample t-test.

b The P value was obtained by chi-square test.

### Differences in resting state FC values between patients with LLD and comparison subjects

**Figure 1:**
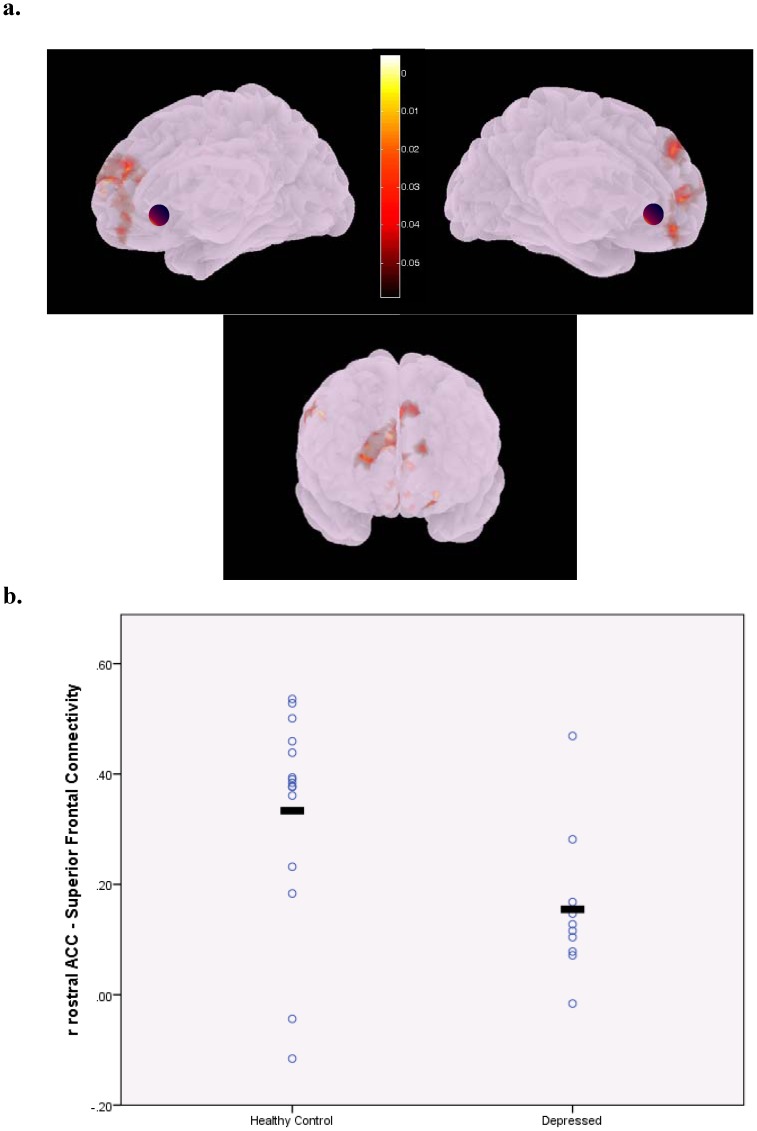
Illustration of areas in the bilateral superior frontal cortex showing decreased connectivity with the right rostral anterior cingulate cortex (indicated by the purple sphere) in unmedicated patients with late-life depression (a). Note these areas encompass the bilateral dorsolateral and dorsomedial prefrontal cortex regions. The colorbar indicates p-values (<.05). Scatterplot displaying group differences in individual functional connectivity values represented by z-scores (FDR corrected p = .006) (b).

**Figure 2:**
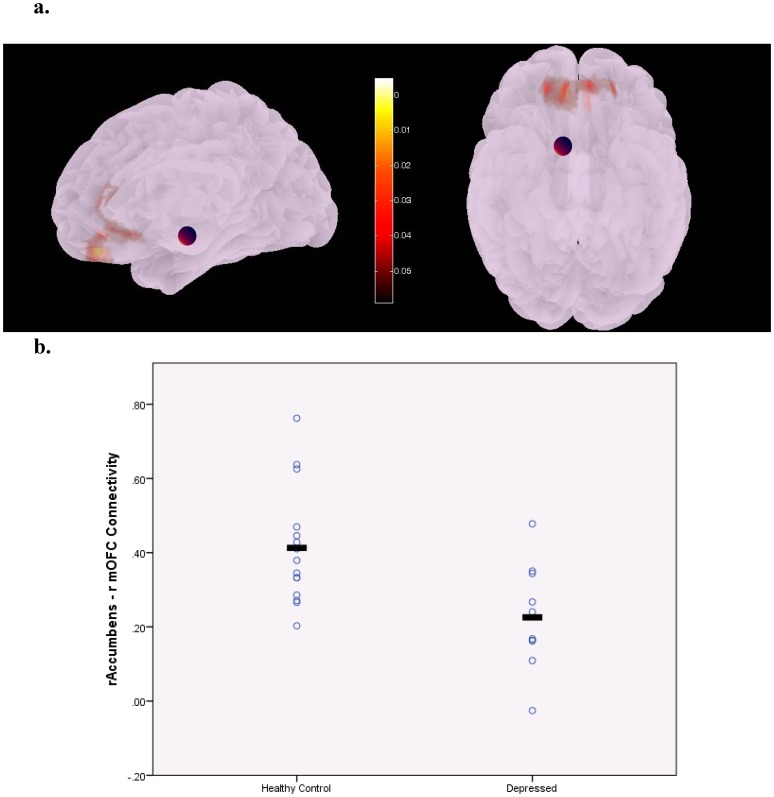
Illustration of areas in the medial orbitofrontal cortex with decreased connectivity with the right accumbens area (indicated by the purple sphere) in unmedicated patients with late-life depression. The colorbar indicates p-values (<.05) (a). Scatterplot displaying group differences in individual functional connectivity values represented by z-scores (FDR corrected p = .035) (b).

Compared to the healthy comparison group, depressed subjects had significantly lower connectivity between the right rostral anterior cingulate cortex (rrACC) and bilateral superior frontal gyrus (bsFG) (Figure 1,connection-level FDR corrected p = 0.006, additional seed-level corrected p = 0.6, partial η^2^ = .53) as well as between the right Accumbens area (rA) and the right medial orbitofrontal cortex (rmOFC) (Figure 2, connection-level FDR corrected p = 0.035, additional seed-level corrected p = 0.95, partial η^2^ = .47). Our results revealed a significant negative correlation between rA-rmOFC connectivity and depression severity across the whole sample (*r* = −0.488; p = 0.034) but not among depressed subjects (*r* = −0.241; p = 0.602). In the whole sample and in the depressed group, a significant negative correlation was also detected between rrACC-bsFG connectivity and GDS score (*r* = −0.704; p = 0.001 for whole sample and *r* = −0.771; p = 0.043 for depressed group).

### FA changes and its correlation with resting state FC changes in LLD

**Figure 3:**
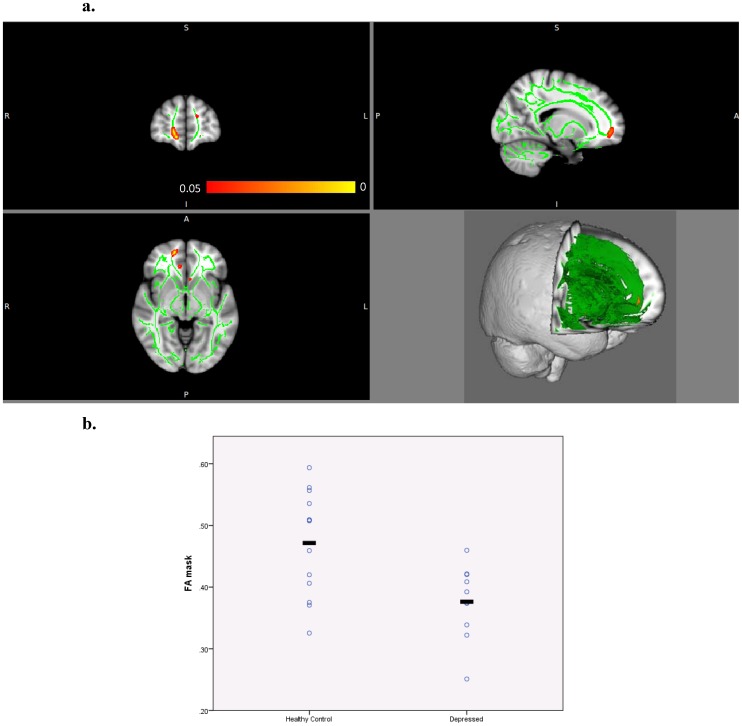
Reduced FA in the right forceps minor (in red-orange) in unmedicated depressed elderly compared to healthy comparison subjects. The mean FA skeleton is shown in green superimposed onto the standard MN152 brain temple (upper left: coronal view, upper right: sagittal, lower left: axial, lower right: 3D view). The colorbar indicates p-values (<.05) (a). Scatterplot displaying group differences in individual FA values for the masked region in the right forceps minor (uncorrected p<0.0005) (b).

**Figure 4:**
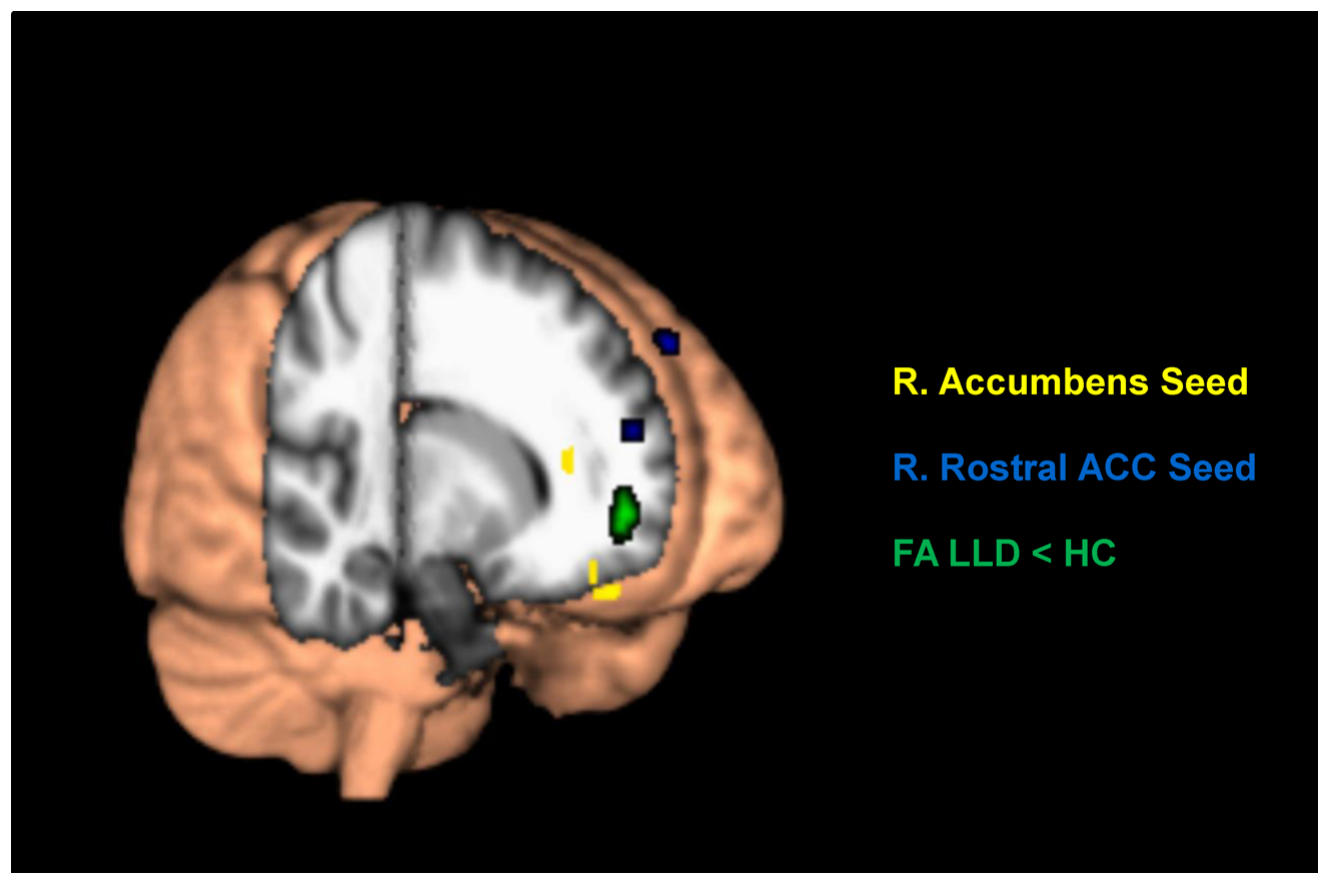
Functional connectivity (FC) and fractional anisotropy (FA) differences indicated on a 3D view of the brain. Regions in yellow represent areas of decreased FC with the right accumbens area, regions in blue indicate areas of decreased FC with the right rostral ACC, and reduced white matter FA in LLD is shown in green.

Our results revealed a 20% reduction in FA in the right Forceps Minor (rFM) in depressed subjects relative to healthy subjects (HC: 0.471±.085, LLD: 0.376±.060, uncorrected p<0.0005) (Figure 3). We found strong positive correlations between rFM FA and rA-rmOFC across total sample (r = 0.623, p = 0.003) and between rFM FA and rrACC-bsFG FC across the total sample (r = 0.627, p = 0.003) and within the depressed subjects group (r = 0.800, p = 0.031). In comparison subjects, our analysis did not show any significant correlation between rFM FA and rA-rmOFC or rrACC-bsFG connectivity. Figure 4 shows the regions of FA alterations relative to the areas of decreased FC.

## Discussion

To the best of our knowledge, this is the first study investigating whole-brain rs-fMRI FC changes and DTI in unmedicated and symptomatic patients with late-life depression. Our data-driven rs-fMRI FC analysis revealed significant decrease in connectivity between the right rostral ACC and bilateral superior frontal gyrus as well as between the right Accumbens area and the right mOFC. TBSS analysis revealed in LLD subjects a significant decrease in the FA value of the right FM hat correlated with observed alterations in FC.

### sFG and rACC interactions in emotion regulation and depression

We found reduced functional connectivity between the right rACC and bilateral sFG in LLD. Based on the results of several functional brain imaging studies in healthy subjects and patients with mood and anxiety disorders, both the sFG and rACC participate in a complex frontolimbic circuit suggested as the neural correlate of emotion regulation. Coactivation of the right sFG and the right rACC was detected during a task that required emotional self-regulation in normal participants [44]. Mies et al reported an increase in activity of the rACC, right sFG and posterior cingulate cortex (PCC) when subjects needed to process the valence of performance feedback in a time-estimation task involving emotion-cognition interaction [45]. In another study in line with our results, Aizenstein et al detected lower DLPFC-dorsal anterior cingulate cortex (dACC) connectivity during performance of an executive-control task in depressed patients in relative to control elderly subjects [14]. Alexopoulos and his colleagues showed a decrease in FC between the dACC and DLPFC and also between dACC and bilateral inferior parietal cortices in patients with LLD in relative to healthy control group [21]. Interestingly, their study revealed low FC of DLPFC with dACC as a significant predictor for depressive symptoms persistence, low remission rate, apathy and dysexecutive behavior in depressed subjects. They also found reduced connectivity of the right nucleus accumbens (rNAcc) and OFC in LLD, which is consistent with our results [26].

The subgenual anterior cingulate cortex (sgACC) is located in the ventral part of rACC. A set of neuropathological, structural and functional brain imaging and animal lesion studies have suggested a strong involvement of the sgACC in depression and mania [46]. PET and fMRI studies have shown increased metabolic activity in the sgACC during depression that correlates with symptoms severity [47], [48]. Furthermore, a decrease in activity of the sgACC was reported in response to various treatment modalities including direct deep brain stimulation (DBS) of sgACC [46], [49]–[51]. The DLPFC, a part of sFG that showed reduced FC with rACC in the present study, has also been implicated in midlife and late-life depression [14], [51], [52]. Interestingly, the pattern of depression-related abnormalities in the DLPFC and sgACC are in opposite directions. Unlike sgACC, hypoactivity in DLPFC has been found during the depressive period [51]. In a TMS/rs-fMRI FC study by Fox et al, better treatment responses were reported by transcranial magnetic stimulation of DLPFC sites with higher anticorrelation with sgACC suggesting the modulatory effect of DLPFC on sgACC for treating depression [53], [54]. In a recent ECT/RS-fMRI FC study, a significant increase in connectivity between the ACC and the right DLPFC was found after ECT treatment of depressed subjects [55]. Interestingly, in that study a strong linear correlation was observed between change of ACC-right DLPFC connectivity and change of depression severity measured by HAM-D scores, which is in line with our results. Based on the detected FC decrease between sFG and rrACC in the present study, we are suggesting impairment in regulatory effect of sFG (especially the DLPFC) on limbic regions like the sgACC as a circuit-based mechanism mediating LLD.

### OFC and NAcc in reward processing and depression

The NAcc and the OFC comprise part of the “reward network” involved in reward processing and hedonic experience. Accumulating findings from neuroimaging, neuropsychology and neurophysiology studies link the OFC to sensory integration, reward value processing, decision making and subjective pleasantness [56]. Structural and functional abnormalities in the lateral posterior and mOFC have been implicated in the pathophysiology of mood disorders [57]. As a key node in reward neurocircuitry, the NAcc is located in the ventral striatum and serves important functional roles in reward and motivational processing, reward-based decision making, learning and hedonic experience [58], [59]. Results of several human brain imaging and animal studies have strongly suggested the involvement of neurochemical and functional disturbances in the NAcc in the genesis of drug seeking behavior and depression [60], [61].

The OFC and medial prefrontal cortex sends direct afferent projections to the NAcc [62]. The NAcc sends its output to the prefrontal cortex indirectly through the ventral pallidum (VP) and the medial dorsal nucleus of the dorsal thalamus. rs-fMRI FC analysis in healthy subjects showed strong FC between the NAcc and the orbitomedial prefrontal cortex (Brodmann areas 11, 13, 24, 25, and 32)[63]. The literature indicates that this FC is impaired in depression. In a recent study by Alexopoulos and colleagues, the FC pattern of the NAcc was investigated in apathetic and non-apathetic patients with LLD [26]. They found FC values of the NAcc with several areas including striatum, prefrontal cortex and insula differed between apathetic and non-apathetic depressed subjects. Consistent with our results, their results also revealed lower FC between the right NAcc and bilateral OFC in typical non-apathetic but depressed subjects compared to a healthy control group. In line with Alexopoulos et al results, our findings suggest functional disturbances in the reward network due to impaired interaction of two key nodes of this circuitry may serve as another network-based mechanism of pathology in LLD.

### Relation between structural and functional connectivity in LLD

In a recent structural/functional connectivity study of adults with MDD, Kwaasteniet et al, showed a negative correlation between uncinate fasciculus integrity and subgenual ACC functional connectivity with the bilateral hippocampus in subjects with MDD compared to a healthy control group. They also found a positive correlation between this reported negative structure-function relationship and depression severity in depressed patients [64]. Only two studies have examined the relationship between white matter integrity and FC in LLD. In one study of Wu et al, a negative correlation was reported between whole brain white-matter hyperintensities burden and resting state connectivity in the medial frontal region in unmedicated elderly patients with LLD [24]. In another study by Steffens et al, uncinate fasciculus (UF) tract was selected as the predefined region of interest for DTI study and positive correlations were found between the left uncinate fasciculus (UF) FA and left ventrolateral PFC-left amygdala as well as left ventrolateral PFC- left hippocampus resting state functional connectivity [30]. In both studies, the structural connectivity- functional connectivity relationship was only investigated in elderly subjects with LLD with no healthy comparison group. The present study extends these finding by integration our FC analysis with TBSS whole brain DTI in unmedicated and symptomatic subjects with LLD and a healthy comparison group.

Interestingly, our main TBSS finding of LLD-related alteration in the rFM is consistent with our FC results. The forceps minor is the anterior extension of corpus callosum in the frontal lobes connecting the lateral and medial regions of frontal lobes and extends to the striatum and limbic areas. It is very likely that the disruption in integrity of this fiber bundle compromises the axonal tracts mediating both bsFG-rACC and OFC-NAcc anatomical connections. This possible white matter structural abnormality can reasonably be suggested as an underlying etiological mechanism for observed FC changes in our depressed subjects and explains the significant correlation between structural and functional connectivity values reported in the present study. However, prospective longitudinal studies are needed to determine causality.

### Limitations and methodological considerations

A limitation of the present study is the small sample size. Despite the small sample size, the results demonstrated a very robust effect size based on the partial η^2^ values. Approximately half the variance in FC values for the rrACC – bilateral SFG and rA– rmOFC was explained by group differences. Another limitation is the use of anatomical ROIs instead of functional ROIs. The use of anatomical ROIs can result in the combination of multiple functionally distinct regions and thus confound our results. Notwithstanding this caveat, there are several methodological strengths to consider. In addition, rather than examine a selected set of predefined brain regions of interest, we applied a comprehensive data-driven connectome method for broadly exploring whole brain FC changes in LLD. Compared to *a priori* hypothesis-based methods, our approach for data analysis was not biased by the choice of seed selection and therefore has higher sensitivity without compromising specificity. Another strength of the present study is our subject selection which involved unmedicated and symptomatic depressed participants. Several studies have shown significant effect of psychotropic medications including SSRIs on brain networks dynamics and FC in depression [65], [66]. Furthermore, the pattern and values of FC were reported to be sensitive and correlated to depression severity [67], [68] as well as altered after symptoms remission in LLD [24]. To limit potential medication-related and remission-related FC confounds, we chose a group of unmedicated (60% were treatment-naïve) and symptomatically depressed subjects for the current study. While the study is limited by the small sample size, the FC findings were all statistically significant after FDR correction for multiple comparisons. Multiple comparison correction was not used for the TBSS analysis since we had an *a priori* region of interest based on the FC findings. However, the uncorrected threshold for significance was set at p = .0005. Future studies expanding the sample size would strengthen the validity of our findings. Another limitation is the relatively older age of our comparison subjects compared to the depressed patients. To address this issue, we carefully corrected all significant group differences for age. Furthermore, it should be noted that the older age in the comparison group would likely have a mitigating effect on our results.

### Conclusion

Based on the findings of the present study, we propose two main circuit-based mechanisms for LLD at the network functional level: impairment in top-down frontolimbic emotion regulation circuitry and frontostriatal reward networks reflected respectively in the associated decrease in bsFG-rrACC and rA-rmOFC connectivity. We found those FC changes serve as significant biological markers for symptom severity in unmedicated and symptomatic subjects with LLD. Our DTI structural imaging results detected a pathologic white matter integrity measure alteration in the rFM that significantly correlated with above-mentioned FC differences and depression severity. This correlation suggests an interesting relationship between structural and functional connectivity in LLD. The association of structure-function alterations with specific cognitive, affective and somatic depressive symptoms will be an important direction for this line of study in the future.
